# Using Radiomics and Explainable Ensemble Learning to Predict Radiation Pneumonitis and Survival in NSCLC Patients Post-VMAT

**DOI:** 10.3390/life15111753

**Published:** 2025-11-14

**Authors:** Tsair-Fwu Lee, Lawrence Tsai, Po-Shun Tseng, Chia-Chi Hsu, Ling-Chuan Chang-Chien, Jun-Ping Shiau, Yang-Wei Hsieh, Shyh-An Yeh, Cheng-Shie Wuu, Yu-Wei Lin, Pei-Ju Chao

**Affiliations:** 1 Medical Physics and Informatics Laboratory of Electronics Engineering, National Kaohsiung University of Science and Technology, No. 415, Jiangong Rd., Sanmin Dist, Kaohsiung 80778, Taiwan; tflee@nkust.edu.tw (T.-F.L.); cclcnunu@gmail.com (L.-C.C.-C.); wewe750422@gmail.com (Y.-W.H.); 2 Graduate Institute of Clinical Medicine, Kaohsiung Medical University, Kaohsiung 80708, Taiwan; 3 Department of Medical Imaging and Radiological Sciences, Kaohsiung Medical University, Kaohsiung 80708, Taiwan; 4 Department of Medical Imaging and Radiological Sciences, I-Shou University, Kaohsiung 82445, Taiwan; sayeh@outlook.com; 5 Department of Radiation Oncology, E-DA Hospital, Kaohsiung 82445, Taiwan; 6 Department of Radiation Oncology, Columbia University, New York, NY 10032, USA; 7 Department of Radiation Oncology, Kaohsiung Veterans General Hospital, Kaohsiung 813, Taiwan; 8 Department of Radiation Oncology, Kaohsiung Chang Gung Memorial Hospital and Chang Gung University College of Medicine, Kaohsiung 83301, Taiwan

**Keywords:** volumetric modulated arc therapy, lung cancer, radiation pneumonitis, survival analysis, radiomics, machine learning, explainable artificial intelligence, ensemble learning

## Abstract

**Purpose**: This study aimed to develop a precise predictive model to assess the risk of radiation pneumonitis (RP) and three-year survival in patients with non-small cell lung cancer (NSCLC) following volumetric modulated arc therapy (VMAT). Radiomics features, ensemble stacking, and explainable artificial intelligence (XAI) were integrated to enhance predictive performance and clinical interpretability. **Materials and Methods**: A retrospective cohort of 221 NSCLC patients treated with VMAT at Kaohsiung Veterans General Hospital between 2013 and 2023 was analyzed, including 168 patients for RP prediction (47 with ≥grade 2 RP) and 118 patients for survival prediction (34 deaths). Clinical variables, dose–volume histogram (DVH) parameters, and radiomic features (original, Laplacian of Gaussian [LoG], and wavelet filtered) were extracted. ANOVA was used for initial feature reduction, followed by LASSO and Boruta-SHAP for feature selection, which formed 10 feature subsets. The data were divided at an 8:2 ratio into training and testing sets, with SMOTE balancing and 10-fold cross-validation for parameter optimization. Six models—logistic regression (LR), random forest (RF), support vector machine (SVM), k-nearest neighbors (KNN), XGBoost, and Ensemble Stacking—were evaluated in terms of the AUC, accuracy (ACC), negative predictive value (NPV), precision, and F1 score. SHAP analysis was applied to interpret feature contributions. **Results**: For RP prediction, the LASSO-selected radiomic subset (FR) combined with Ensemble Stacking achieved optimal performance (AUC 0.91, ACC 0.89), with SHAP identifying V40 Firstorder_Min as the most influential feature. For survival prediction, the FR subset yielded an AUC of 0.97, an ACC of 0.92, and an NPV of 1.00, with V10 Wavelet Firstorder_Min as the top contributor. The multimodal subset (FC+R) also performed strongly, achieving an AUC of 0.91 for RP and 0.96 for survival. **Conclusions**: This study demonstrated the superior performance of radiomics combined with Ensemble Stacking and XAI for the prediction of RP and survival following VMAT in patients with NSCLC. SHAP-based interpretation enhances transparency and clinical trust, offering a robust foundation for personalized radiotherapy and precision medicine.

## 1. Introduction

Lung cancer is one of the leading cancers worldwide. According to the International Agency for Research on Cancer (IARC) in 2022, it accounts for approximately 12% of all cancer cases and 18% of cancer-related deaths [1], with non-small cell lung cancer (NSCLC) accounting for approximately 85% of cases [2]. Volumetric modulated arc therapy (VMAT) has improved treatment efficiency and dose conformity in NSCLC; however, radiation pneumonitis (RP) remains one of the most common complications, with an incidence of 22–28.6% [3,4,5]. RP can cause significant lung function impairment and reduce patients’ quality of life. Accurate prediction of RP risk and three-year survival at an early treatment stage would allow for optimized treatment strategies [6], reducing complications and overtreatment while improving survival outcomes [7]. This provides the rationale for developing precise predictive models that integrate radiomics, ensemble learning, and explainable artificial intelligence (XAI) to advance individualized therapy in NSCLC.

Radiomics extracts high-dimensional quantitative features, such as shape, intensity, and texture, from CT images [8], offering new opportunities for RP and survival prediction in NSCLC [9]. Recent studies have shown that applying Laplacian of Gaussian (LoG) and wavelet filters enhances edge and texture features, improving sensitivity [10]. Advances in artificial intelligence (AI), particularly machine learning (ML), further enable efficient analysis of multidimensional data. For example, deep learning–based radiomic models that integrate dosimetric and imaging features have demonstrated improved RP prediction. Ensemble learning methods, such as stacking, combine predictions from multiple models to reduce bias and improve generalizability and have recently shown advantages in lung cancer risk assessment. However, the “black-box” nature of ML models limits their clinical applicability [11]. XAI techniques, such as SHAP (SHapley Additive exPlanations) [12], enhance interpretability by quantifying feature contributions. While most prior studies focused on single modalities or lacked interpretability, our study integrates multimodal features (clinical, dose-volume histogram (DVH), and radiomics) with XAI to overcome these limitations and advance precision medicine.

Traditional RP and survival prediction methods rely on normal tissue complication probability (NTCP) models [13], which are primarily based on the mean lung dose (MLD) and Vdose parameters derived from DVH [14]. These models often ignore clinical variables and imaging heterogeneity [15], leading to limited accuracy (approximately 75%). Recent evidence further suggests that single-modality models cannot adequately capture microstructural lung changes. For example, Su et al. (2024) [16] proposed a multiomics deep learning model that integrates dosiomics but did not address interpretability. Tang et al. (2025) [17] developed a dual-modal fusion model that improved RP prediction but lacked transparency. Wang et al. (2025) [18] also highlighted that clinical variables alone are insufficient to distinguish RP, necessitating radiomics integration. Against this backdrop, our study leverages radiomics with LoG and wavelet filters to capture imaging heterogeneity combined with XAI to enhance interpretability, thereby achieving predictive performance comparable to or exceeding that of recent multimodal models.

The objective of this study was to develop a precise predictive model to evaluate RP risk and three-year survival in NSCLC patients treated with VMAT. By integrating clinical, DVH, and radiomic features, we constructed Ensemble Stacking models optimized for performance and applied SHAP analysis to identify key feature contributions. The study specifically focused on identifying imaging features within dose regions (e.g., V40, V10) relevant to prediction, providing clinicians with reliable prognostic insights to optimize individualized treatment strategies, reduce RP risk, and improve survival outcomes.

## 2. Materials and Methods

### 2.1. Study Design

This study aimed to predict the risk of RP and three-year survival in patients with NSCLC treated with VMAT. A multimodal framework was developed by integrating clinical variables, DVH parameters, and radiomic features extracted from pretreatment chest CT images. Radiomics features were initially reduced via analysis of variance (ANOVA), followed by the construction of ten single- and multimodal feature subsets: clinical features (FC), DVH features (FDVH), original radiomic features (FR_O), Laplacian of Gaussian (LoG)–filtered radiomic features (FR_LoG), wavelet-filtered radiomic features (FR_W), combined radiomic features (FR), and multimodal combinations (FC+DVH, FC+R, FDVH+R, and FC+DVH+R).

**Figure 1:**
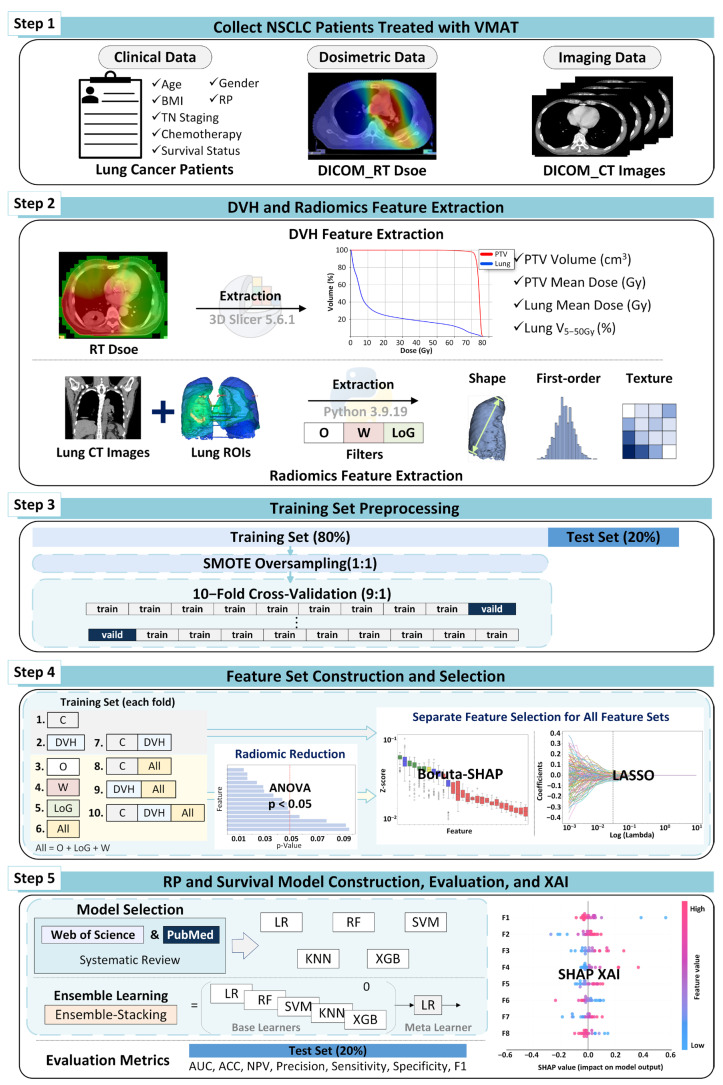
Overall study workflow segmented into five steps (Step 1–5) for improved readability. **Step 1:** Data acquisition (clinical, dosimetric, imaging); **Step 2:** Feature extraction (DVH, radiomics with filters); **Step 3:** Preprocessing (SMOTE oversampling); **Step 4:** Feature selection (ANOVA, Boruta-SHAP, LASSO); **Step 5:** Model construction, evaluation, and XAI (Ensemble Stacking, metrics, SHAP). The figure annotates the fold-wise preprocessing pipeline, indicating that ANOVA filtering, LASSO/Boruta-SHAP feature selection, and SMOTE oversampling were performed independently for each training fold during cross-validation, with the test set evaluated only once after model training. Abbreviations: VMAT, Volumetric Modulated Arc Therapy; BMI, Body Mass Index; RP, Radiation Pneumonitis; TN, Tumor Node Staging; DICOM, Digital Imaging and Communications in Medicine; RT, Radiation Therapy; CT, Computed Tomography; DVH, Dose-Volume Histogram; Gy, Gray; PTV, Planning Target Volume; ROI, Region of Interest; SMOTE, Synthesized Minority Oversampling Technique; O, Original image; W, Wavelet; LoG, Laplacian of Gaussian; ANOVA, Analysis of Variance; LASSO, Least Absolute Shrinkage and Selection Operator; LR, Logistic Regression; RF, Random Forest; SVM, Support Vector Machine; KNN, K-Nearest Neighbors; XGBoost, eXtreme Gradient Boosting; AUC, Area Under the ROC curve; ROC, Receiver Operating Characteristic; ACC, Accuracy; NPV, Negative Predictive Value; F1, F1-score; SHAP, SHapley Additive exPlanations.

Feature selection was performed via Boruta-SHAP and LASSO to identify the most informative predictors. LASSO was implemented using scikit-learn (version 1.5.1, Python 3.9), while Boruta-SHAP was implemented using the publicly available Python package (version 1.0.20). The predictive models included logistic regression (LR), random forest (RF), support vector machine (SVM), k-nearest neighbors (KNN), XGBoost, and Ensemble Stacking. Model performance was evaluated using the area under the curve (AUC), accuracy (ACC), negative predictive value (NPV), precision, sensitivity, specificity, and F1 score. Finally, SHAP analysis was applied to interpret feature contributions and enhance model transparency. The overall workflow of the study is illustrated in Figure 1.

### 2.2. Patient Cohort

This retrospective study included 221 patients with histologically confirmed NSCLC who received VMAT at Kaohsiung Veterans General Hospital between January 2013 and June 2023. The inclusion criteria were as follows: (1) pathologically confirmed NSCLC; (2) a prescribed radiation dose of 50–70 Gy; and (3) availability of complete clinical, imaging, and treatment records. The exclusion criteria included incomplete data, metastatic lung cancer, or concurrent malignancies.

For RP prediction analysis, 168 patients were eligible, of whom 47 (28%) developed ≥grade 2 RP. For survival prediction, 118 patients with at least three years of follow-up data were included, and 34 death events were recorded (three-year survival rate: 71%). RP was graded according to RTOG toxicity criteria on the basis of clinical symptoms and chest CT follow-up evaluations. Initial toxicity assessments were performed by an attending radiation oncologist and independently reviewed by a thoracic radiologist; any discrepancies were resolved through multidisciplinary consensus to ensure grading consistency.

Data were retrieved from electronic medical records and TPS archives, anonymized, and recoded following IRB approval (approval number: KSVGH25-CT1-06; approval date: 17 December 2024). Simulation CT images were acquired using a GE Discovery CT590 RT scanner (GE Healthcare, Waukesha, WI, USA; 512 × 512 matrix, 2.5 mm slice thickness). Treatment planning was performed using Pinnacle^3^ v9.14 (Philips Healthcare, Fitchburg, WI, USA) or Eclipse v13 (Varian Medical Systems, Palo Alto, CA, USA), and radiation was delivered using Elekta Synergy or Versa HD linear accelerators (Elekta AB, Stockholm, Sweden). The materials used in this study included DICOM CT images, RT dose files, and clinical parameters.

### 2.3. VMAT Treatment

VMAT delivers radiation through dynamic modulation of the beam intensity [19], multileaf collimator (MLC) position, and dose rate, achieving conformal dose distribution through inverse planning. The optimization objective minimized deviations from the prescribed tumor dose while limiting exposure to normal tissue. Patients received 50–70 Gy in 2-Gy fractions. The workflow included (1) CT imaging, (2) contouring of targets and organs at risk (OARs), (3) dose calculation and verification, and (4) treatment delivery via 360° arc therapy.

### 2.4. Outcome Definition

RP was defined as symptomatic pulmonary inflammation following radiotherapy and was characterized by radiographic features such as ground-glass opacities. RP was graded according to the RTOG criteria [20], with ≥grade 2 (requiring medical intervention) being defined as RP positive. RP was assessed by radiation oncologists, who reviewed CT scans and clinical symptoms within six months post-treatment.

Survival was defined as three-year overall survival, with deaths occurring within three years considered events.

### 2.5. ROI Delineation

The bilateral lungs were segmented via automated contouring tools in the clinical TPS (Pinnacle^3^ v9.14 or Eclipse v13), followed by manual delineation of the gross tumor volume (GTV) by a board-certified radiation oncologist in accordance with institutional protocols. The final region of interest (ROI; Lungs–GTV) was generated via Boolean subtraction to exclude tumor regions. All contours were subjected to peer review by a second senior radiation oncologist to ensure consistency and minimize interobserver variation.

### 2.6. Feature Extraction

DVH features: Seven regions of interest (ROIs) were delineated, including the planning target volume (PTV) and lung subvolumes (V5–V50 Gy) [21]. The RT dose files were imported into 3D Slicer (v5.6.1) for DVH calculation, yielding nine features: PTV, PTV mean dose, lung mean dose, and lung V5–V50 percentages.

Radiomics features: Features were extracted via PyRadiomics (v3.1.0, Python 3.9) in compliance with IBSI standards [22]. ROIs were processed with original, LoG (sigma = 1.0–3.0 mm), and wavelet filters (8 subbands). For each ROI, 105 features were extracted, including 14 shape, 18 first-order, and 73 texture features (GLCM, GLSZM, GLRLM, NGTDM, GLDM). Across all the ROIs and preprocessing methods, 8379 features were obtained [23].

### 2.7. Feature Selection

Feature selection followed three stages: (1) ANOVA filtering (*p* < 0.05) reduced the number of radiomic features to 615 for RP and 1171 for survival; (2) Boruta-SHAP wrapper selection on the basis of feature contribution; and (3) LASSO embedded selection for penalized regression [24]. Ten feature subsets were generated and analyzed (Supplementary Tables S1 and S2).

To compare model performance between different feature selection methods (e.g., LASSO vs. Boruta-SHAP), DeLong’s test was used to assess the statistical significance of AUC differences, accounting for correlated predictions within the same test set. McNemar’s test was applied to compare the ACC, with a focus on paired differences in classification outcomes.

### 2.8. Literature-Guided Model Choice

A systematic literature review was performed via PubMed and Web of Science [25], guided by PICO-based keywords [26]. Relevant studies were screened and filtered, with selected references supporting the inclusion of LR, RF, SVM, KNN, and XGBoost as base learners for Ensemble Stacking.

### 2.9. Ensemble Stacking

**Figure 2:**
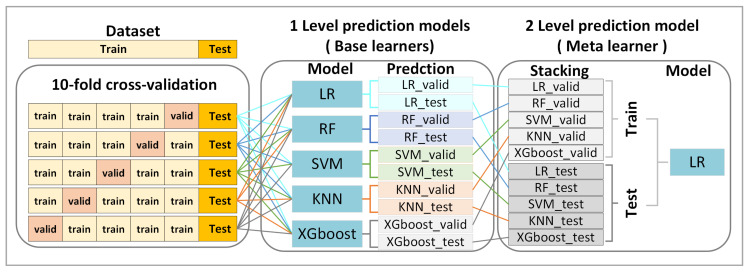
Workflow of the ensemble-stacking model for classification. Abbreviations: LR, Logistic Regression; RF, Random Forest; KNN, K-Nearest Neighbors; SVM, Support Vector Machine; XGBoost, eXtreme Gradient Boosting.

The Ensemble Stacking framework consists of two layers (Figure 2). The base learners (LR, RF, SVM, KNN, and XGBoost) generated predictions, which were then combined by a meta-learner (LR). Predictions from base models (P_base1 … P_base5) served as inputs to the meta-model, reducing bias and improving generalizability.

### 2.10. Model Training and Evaluation

**Figure 3:**
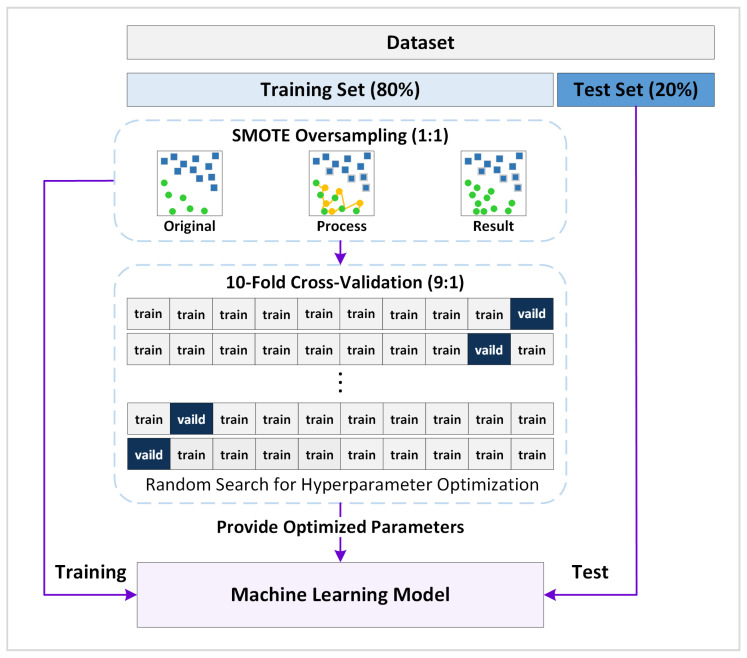
Flowchart of machine learning model construction, training, validation, and testing. Abbreviation: SMOTE, Synthesized Minority Oversampling Technique.

To ensure robust internal validation and prevent data leakage, the dataset (n = 221; 168 for RP prediction and 118 for survival prediction) was randomly split at an 8:2 ratio into training (80%) and test (20%) sets via stratified sampling to maintain class balance between positive and negative outcomes (Figure 3). Within the training set, 10-fold cross-validation was performed, during which ANOVA filtering (*p* < 0.05), LASSO/Boruta-SHAP feature selection, and SMOTE oversampling were applied independently within each cross-validation fold to optimize model performance and address class imbalance (47 RP events and 34 deaths).

SMOTE was implemented via scikit-learn (v1.5.1) with the following parameters: k_neighbors = 5, sampling_strategy = “auto”, and random_state = 42, ensuring preservation of the underlying feature distribution while augmenting minority class samples. To further prevent data leakage and synthetic data bias, all preprocessing—including feature selection and oversampling—was performed strictly within the training folds, whereas the independent test set remained completely isolated from any preprocessing or tuning procedures.

The final model was retrained on the complete training set via the optimized pipeline and evaluated on the independent test set via the AUC, accuracy, NPV, precision, sensitivity, specificity, and F1 score. External validation using independent datasets is needed to confirm generalizability.

### 2.11. SHAP Analysis

SHAP (SHapley Additive exPlanations) was applied to the best-performing models to quantify feature contributions [27]. Beeswarm plots were generated, ranking the top 20 features. Each point represents a feature instance, colored by feature value (blue–red), with the *x*-axis representing SHAP values. This analysis enhanced model interpretability and provided additional clinical insight.

## 3. Results

### 3.1. Patient Clinical Characteristics

A total of 168 NSCLC patients treated with VMAT were included for RP prediction (incidence 28%). As shown in Table 1, no significant differences were observed between the RP (n = 47) and non-RP (n = 121) groups in terms of age (mean 69.6 vs. 67.8 years), BMI (mean 24.4 vs. 23.4), sex, T/N stage, or chemotherapy status (all *p* > 0.05). These findings indicate that clinical variables alone were insufficient to distinguish RP risk, underscoring the necessity of integrating radiomics and dosimetric features for accurate prediction.

For survival prediction, 118 patients were analyzed (three-year survival rate: 71%). As shown in Table 2, the survival (n = 84) and death (n = 34) groups did not significantly differ in age, sex, T/N stage, or chemotherapy status, except for BMI, which was significantly greater in survivors (24.1 vs. 22.0, *p* = 0.022). These findings suggest that an elevated BMI may be associated with improved survival, highlighting its potential as a prognostic biomarker that may reflect nutritional status or metabolic characteristics relevant to long-term outcomes.

### 3.2. Feature Extraction and Selection

The feature sources included seven clinical variables (FC), nine DVH features (FDVH), and 8379 radiomic features (FR) derived from original CT, LoG-filtered, and wavelet-filtered images. After ANOVA-based filtering, radiomic subsets were formed and further reduced by Boruta-SHAP and LASSO.

For RP prediction, 615 radiomics features were retained after ANOVA. LASSO selects 32–35 features, outperforming Boruta-SHAP (15–18 features). Notably, wavelet-derived features (442) dominated RP prediction, capturing subtle textural heterogeneity in lung tissue that reflects microstructural damage.

For survival prediction, 1171 radiomic features were retained, with LASSO selecting 24–28 and Boruta-SHAP selecting 16–19 features. Wavelet features (778) again contributed substantially, whereas multimodal subsets (e.g., FDVH+R) further enriched predictive diversity. These results emphasize the superiority of radiomics in both RP and survival prediction, particularly for wavelet-derived textural features that capture tumor heterogeneity and microenvironmental variations beyond conventional clinical and DVH metrics.

### 3.3. Model Performance Evaluation

Six models (LR, RF, SVM, KNN, XGBoost, and Ensemble Stacking) were constructed and evaluated in terms of the AUC, ACC, NPV, precision, sensitivity, specificity, and F1 score. As summarized in Table 3 and Supplementary Tables S3–S6, LASSO-based feature selection consistently outperformed Boruta-SHAP, with Ensemble Stacking achieving the best overall performance.

For RP prediction, Ensemble Stacking using the radiomics subset (FR) achieved the highest performance (AUC 0.91, ACC 0.89, NPV 1.00, F1-score 0.83), far surpassing clinical (FC: AUC 0.68) and DVH (FDVH: AUC 0.54) subsets. Wavelet features alone also demonstrated strong predictive ability (AUC 0.70). These findings highlight the crucial role of radiomics, particularly image filtering techniques, in enhancing RP risk stratification and guiding individualized treatment planning. Statistical comparisons using DeLong’s test confirmed that the LASSO-selected Ensemble Stacking model (AUC 0.91 for RP, 0.98 for survival) significantly outperformed Boruta-SHAP-selected models (AUC 0.87 for RP, 0.94 for survival; *p* < 0.05). Similarly, McNemar’s test indicated significant improvements in ACC for LASSO-selected models (ACC 0.88 for RP, 0.96 for survival) compared to Boruta-SHAP (*p* < 0.05).

For survival prediction, Ensemble Stacking using the FR subset achieved the best performance (AUC 0.97, ACC 0.92, NPV 1.00, F1-score 0.86), with multimodal subsets such as FC+R also performing well (AUC 0.96). The relatively small number of positive events (n = 47 for RP and n = 34 for death) relative to the high-dimensional feature space raises concerns regarding potential overfitting, despite the rigorous feature selection and validation strategies applied.

The exceptionally high NPV for survival prediction (NPV = 1.00) indicates that no false negatives were observed in the test set, which is clinically valuable for identifying low-risk patients. However, the high-performance metrics, including an AUC of 0.97 and an NPV of 1.00 for survival prediction, should be interpreted with caution, as they may be influenced by the modest test set size (n = 24 for survival) and cohort-specific characteristics. External validation using larger, independent datasets is essential to confirm the robustness and generalizability of these findings.

The observation that RP occurrence was associated with improved three-year survival (86% vs. 65%, *p* = 0.024) is notable. While seemingly paradoxical, this may reflect radiotherapy-induced immune activation, as suggested by Liu et al. (2023) [28]. Radiotherapy can remodel the tumor microenvironment (TME) via cGAS–STING/I-type interferon signaling, enhancing tumor neoantigen presentation, dendritic cell maturation, CD8^+^ T-cell infiltration, and M1 macrophage polarization. These immune responses may link mild RP to improved survival, acting as a surrogate of therapeutic efficacy rather than a causal factor. This finding aligns with that of Niu et al. (2023) [29], who reported better survival in patients with RP.

### 3.4. Feature Importance Analysis

SHAP analysis was conducted to enhance model interpretability.

**Figure 4:**
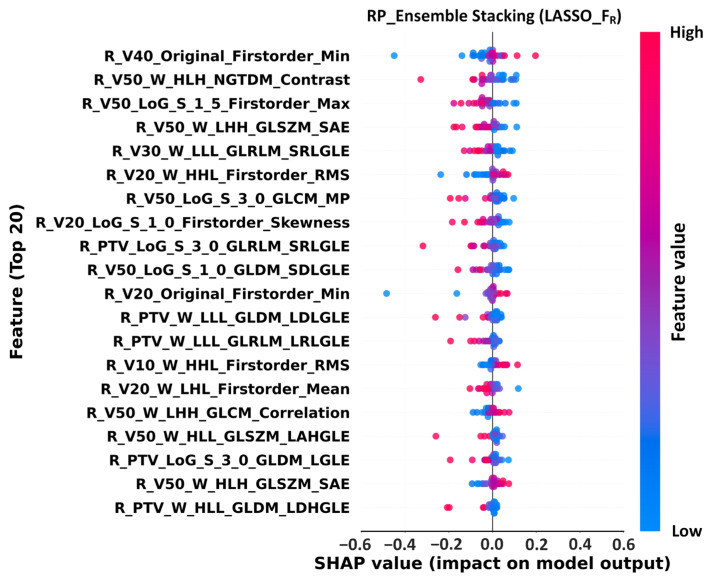
SHAP beeswarm plot for RP prediction using the LASSO-selected radiomics subset (FR) in the Ensemble Stacking model. Each dot represents a feature contribution for a sample; red dots indicate positive SHAP values (higher risk of RP), blue dots indicate negative values (lower risk). Abbreviations: RP, Radiation Pneumonitis; VMAT, Volumetric Modulated Arc Therapy; NSCLC, Non-Small Cell Lung Cancer; LASSO, Least Absolute Shrinkage and Selection Operator; SHAP, SHapley Additive exPlanations; FR, Filtered Radiomics; R, Radiomics; F, Feature; LoG, Laplacian of Gaussian; LoG−Sigma−1−5 mm−3D, Laplacian of Gaussian filter with sigma 1.5 mm in 3D; W, Wavelet; Wavelet-HLH, Wavelet filter in HLH direction; V40, volume receiving ≥ 40 Gy; GLCM, Gray Level Co-occurrence Matrix; GLRLM, Gray Level Run Length Matrix; GLSZM, Gray Level Size Zone Matrix; GLDM, Gray Level Dependence Matrix; NGTDM, Neighboring Gray Tone Difference Matrix; Firstorder_Min, minimum voxel intensity (first-order statistics); Contrast, NGTDM contrast feature; ZonePercentage, GLSZM zone percentage feature; SAE, Small Area Emphasis; SRLGLE, Short Run Low Gray Level Emphasis; SDLGLE, Small Dependence Low Gray Level Emphasis; LDLGLE, Large Dependence Low Gray Level Emphasis; LRLGLE, Long Run Low Gray Level Emphasis; LAHGLE, Large Area High Gray Level Emphasis; LDHGLE, Large Dependence High Gray Level Emphasis; MP, Maximum Probability; RMS, Root Mean Squared.

For RP prediction, the best-performing model (LASSO + FR + Ensemble Stacking) identified Original V40 Firstorder_Min and Wavelet V50 NGTDM_Contrast as the most influential features (Figure 4). High feature values (red points) were positively associated with RP risk, underscoring the critical role of texture heterogeneity in high-dose regions as a driver of RP development.

**Figure 5:**
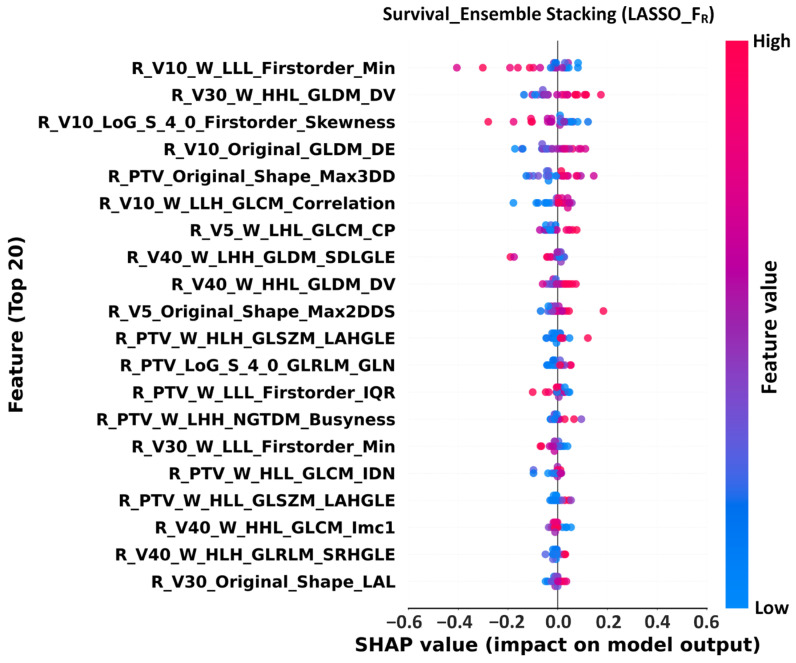
SHAP beeswarm plot for survival prediction using the LASSO-selected radiomics subset (FR) in the Ensemble Stacking model. Each dot represents a feature contribution for a sample; red dots indicate positive SHAP values (higher risk of death), blue dots indicate negative values (better survival). Abbreviations: LASSO, Least Absolute Shrinkage and Selection Operator; FR, Filtered Radiomics; R, Radiomics; SHAP, SHapley Additive exPlanations; W, Wavelet; LoG, Laplacian of Gaussian; LoG-Sigma-1-0 mm-3D, Laplacian of Gaussian filter with sigma 1.0 mm in 3D; V10, volume receiving ≥10 Gy; Firstorder_Min, minimum voxel intensity (first-order statistics); Skewness, distribution skewness; GLCM, Gray Level Co-occurrence Matrix; Maximum Probability, GLCM-based feature; GLRLM, Gray Level Run Length Matrix; GLSZM, Gray Level Size Zone Matrix; GLDM, Gray Level Dependence Matrix; NGTDM, Neighboring Gray Tone Difference Matrix; SDLGLE, Small Dependence Low Gray Level Emphasis; SRHGLE, Short Run High Gray Level Emphasis; LAHGLE, Large Area High Gray Level Emphasis; DV, Dependence Variance; DE, Dependence Entropy; Max3DD, Maximum 3D Diameter; Max2DDS, Maximum 2D Diameter Slice; GLN, Gray Level Non-uniformity; IQR, Interquartile Range; LAL, Least Axis Length.

For survival prediction, the best-performing model (LASSO + FR + Ensemble Stacking) identified Wavelet V10 Firstorder_Min and V30 GLDM_DependenceVariance as the most important features (Figure 5). Low feature values (blue points) negatively impact survival, indicating that density variation in low-dose regions is closely associated with tumor heterogeneity and poor outcomes.

These findings highlight the value of SHAP in providing transparency, validating radiomics as a key prognostic biomarker, and supporting its application in individualized risk stratification and therapeutic decision-making.

## 4. Discussion

This study successfully developed predictive models for radiation pneumonitis (RP) and three-year survival in NSCLC patients treated with VMAT by leveraging radiomic features, ensemble stacking, and explainable artificial intelligence (XAI). Radiomics-based models, particularly those selected by LASSO and combined with Ensemble Stacking, consistently outperformed the clinical and DVH-only models. Importantly, SHAP analysis revealed the biological importance of key radiomic features derived from specific dose regions, thereby improving interpretability and clinical credibility.

For RP prediction, the LASSO-selected FR subset with Ensemble Stacking achieved optimal performance (AUC 0.91, ACC 0.89). SHAP identified Radiomics_V40_Firstorder_Min as the top contributing feature, indicating that the minimum gray-level intensity in the V40 region was strongly associated with RP risk. For survival prediction, the FR subset again yielded the best performance (AUC 0.97, ACC 0.92, NPV 1.00), with Wavelet V10 Firstorder_Min emerging as the most influential feature. These findings suggest that density variations in low-dose regions play a critical role in long-term survival outcomes. Collectively, these findings underscore the strength of radiomics in capturing microstructural heterogeneity in both lung tissue and tumors, whereas XAI provides a transparent link between features and model outputs, thus bridging the gap between AI and clinical practice.

Compared with recent methods, our model demonstrated competitive or superior performance against state-of-the-art (SOTA) methods. For RP prediction, our AUC of 0.91 surpassed that of the deep-learning MergeNet model by Lee et al. (2024) [30], which integrated pretreatment chest CT, clinical, and laboratory data but yielded a lower AUC (~0.69). It also outperformed the multiomics deep learning model by Su et al. (2024) [16] (AUC ~0.90), which lacked explainability, and matched the dual-modal fusion model by Tang et al. (2025) [17] (AUC ~0.88–0.90). For survival prediction, our AUC of 0.97 exceeded that of the AI-based multimodal survival model by Yuan et al. (2025) [31] (AUC ~0.85–0.90) and substantially outperformed the dual-radiomics SOTA model by Zhang et al. (2024) [32], which achieved an AUC = 0.81 ± 0.04. While Su et al. (2024) [16] attained similar performance (AUC 0.95–0.98), our model uniquely integrates SHAP-based interpretability, enhancing clinical transparency and trust. These SOTA comparisons confirm the robustness and clinical relevance of our explainable radiomic–ensemble framework.

The clinical significance of BMI also emerged as an important finding. Consistent with the “obesity paradox” reported by Nitsche et al. (2022) [33], higher BMI was associated with improved survival in our cohort (*p* = 0.022). These findings support BMI as a simple yet valuable prognostic biomarker. Additionally, Supplementary Table S7 revealed a paradoxical association between RP occurrence and improved survival (86% vs. 65%, *p* = 0.024). Although counterintuitive, these findings may indicate that RP reflects treatment sensitivity or immune activation, which aligns with the findings of Niu et al. (2023) [29], who reported a survival benefit in patients who underwent RP. These results suggest that RP should not be viewed solely as a complication but may also serve as a surrogate marker of therapeutic efficacy, warranting further mechanistic investigation.

The feature importance analysis provided further insights. Supplementary Table S8 highlights LoG-based skewness and Wavelet-based GLSZM_SmallAreaLowGrayLevelEmphasis as top RP predictors, emphasizing the role of high-dose region heterogeneity (e.g., V50) in RP risk. Figure 4 confirmed that Original V40 Firstorder_Min and Wavelet V50 NGTDM_Contrast contributed most strongly to RP prediction, reinforcing the relevance of high-dose textural heterogeneity. For survival prediction, Figure 5 shows that Wavelet V10 Firstorder_Min and GLDM_DependenceVariance are critical determinants, highlighting the prognostic importance of low-dose regional heterogeneity. These findings support Demircioğlu (2022) [34], who demonstrated that advanced image preprocessing enhances predictive performance and provides practical guidance for radiotherapy planning to mitigate toxicity while optimizing therapeutic benefit. The radiomic features, integrated with SHAP analysis and AI-driven decision-making tools, offer actionable biomarkers for clinical workflows, supporting personalized radiotherapy planning in NSCLC patients.

SHAP analysis identified key radiomic features (e.g., V40 Firstorder_Min for RP and V10 Wavelet Firstorder_Min for survival) that, while not intuitively actionable, can be integrated into clinical workflows via AI-driven tools embedded in treatment planning systems (TPSs). V40 Firstorder_Min guides dose adjustments to minimize RP risk, and V10 Wavelet Firstorder_Min supports patient stratification for tailored treatment intensification, leveraging SHAP’s interpretable insights to enhance decision-making and precision in NSCLC radiotherapy planning.

Despite the strengths of our radiomics-based Ensemble Stacking framework, several limitations must be acknowledged. This single-center retrospective study included a relatively small sample size (n = 168 for RP and n = 118 for survival), with limited positive outcome cases (n = 47 for RP and n = 34 for death), which may restrict the statistical power and generalizability of the findings. Although the 8:2 training–test split and 10-fold cross-validation with independent preprocessing per fold provided robust internal validation, the lack of external validation on an independent multicenter dataset remains a key limitation. The high predictive performance observed in this study, including an AUC of 0.98 and an NPV of 1.00 for survival prediction, should therefore be interpreted with caution, as these results may reflect cohort-specific characteristics and the limited test set size. The paradoxical association between RP occurrence and improved survival (*p* = 0.024) suggests that mild RP may serve as a surrogate marker of immune activation or treatment sensitivity, as radiotherapy can induce TME remodeling through inflammatory and immune responses [28,29]. However, this finding requires further mechanistic exploration to elucidate the underlying biological pathways, such as the cGAS–STING signaling pathway, and to confirm its clinical implications through prospective studies. The use of DeLong’s and McNemar’s tests to compare model performance (e.g., LASSO vs. Boruta-SHAP) strengthens the robustness of our findings, although the results remain limited by the single-center design and modest sample size. To enhance transparency and reproducibility, Supplementary Tables S1, S2, and S8  detail the feature reduction process, the numbers retained at each stage (ANOVA, Boruta-SHAP, LASSO), and the top selected features, addressing the complexity of the feature selection pipeline.

This paradoxical RP–survival association may reflect radiation-induced immune activation, as radiotherapy remodels the TME via cGAS–STING/I-type interferon signaling, enhancing tumor neoantigen presentation, dendritic cell maturation, and CD8^+^ T-cell infiltration [34]. These findings align with those of Niu et al. (2023) [29], suggesting that mild-to-moderate RP indicates increased tumor immunogenicity and radiosensitivity, serving as a biomarker of treatment response rather than solely a side effect.

The excellent performance of the Ensemble Stacking model (AUC > 0.9) raises concerns about potential overfitting, particularly with a high-dimensional radiomic dataset and modest sample size. However, rigorous safeguards such as the 80:20 stratified train–test split, independent preprocessing within 10-fold cross-validation, LASSO regularization, and evaluation on an untouched test set provide evidence of robustness. Future external validation will further confirm the model’s generalizability.

The modest sample size (n = 168 for RP, n = 118 for survival) may limit statistical power and generalizability, despite the use of SMOTE-facilitated stratification in the 8:2 train–test split to ensure balanced class distribution. Future studies with larger, multicenter cohorts are needed to validate these findings and enhance their clinical applicability. A key limitation of this study is its single-center, retrospective design, which may reduce the generalizability of the predictive model. However, the consistent treatment protocols and imaging parameters used in this cohort ensure robust internal validity. Future work will focus on external validation using multicenter datasets, coupled with image standardization procedures, to enhance model reproducibility and clinical applicability. The use of SMOTE to address class imbalance in the training set may introduce synthetic data bias, potentially affecting model performance. However, its application was restricted to training folds within 10-fold cross-validation to minimize bias, and future studies will explore alternative balancing techniques and larger cohorts to further mitigate this risk.

Additionally, variability from manual GTV delineation cannot be completely excluded, as a formal interobserver variability analysis (e.g., Dice similarity coefficient) was not conducted; however, contour consistency was maintained through expert peer review by a senior radiation oncologist. Class imbalance requiring SMOTE correction, potential underestimation of subclinical RP cases via RTOG criteria, and the computational burden of radiomics extraction and SHAP analysis may also impact model reproducibility and clinical implementation. Future work will focus on expanding the dataset, incorporating multicenter external validation, and developing a simplified pipeline to enhance clinical feasibility.

Although classical machine learning methods such as ANOVA, LASSO, and Ensemble Stacking were employed, the novelty of this study lies in the development of a rigorously validated and clinically interpretable AI framework tailored for VMAT-treated NSCLC patients. Unlike prior studies that relied on single-modality prediction or noninterpretable deep learning approaches, our framework integrates filtered radiomic features (LoG and wavelet), DVH metrics, and clinical variables within an ensemble stacking architecture to enhance both predictive stability and model robustness. Moreover, the incorporation of SHAP-based explainability overcomes the common “black-box” limitation of AI models by providing feature-level transparency. This enabled the identification of dose–region–specific radiomic biomarkers—such as V40 Firstorder_Min for RP risk and V10 Wavelet Firstorder_Min for survival—which establish quantitative links between pulmonary heterogeneity and the radiation response. These findings not only improve predictive performance but also offer actionable value for clinical decision support in personalized radiotherapy planning, advancing the clinical translation of explainable AI in radiation oncology.

To translate this model into a real-time clinical decision support system, a roadmap includes (1) optimizing the model through pruning or lightweight architectures to reduce computational complexity; (2) precomputing radiomic features during treatment planning; (3) leveraging cloud-based computing for intensive tasks such as image filtering and SHAP analysis; (4) integrating with TPS for seamless workflows; and (5) conducting pilot studies to validate real-time performance and clinical utility. Recent 2024–2025 literature on explainable AI in radiation oncology further supports our framework’s impact, including studies on XAI for radiation therapy prediction [35], prediction of radiotherapy side effects via XAI [36], leveraging knowledge for XAI in personalized cancer treatment [37], and practical reviews of AI in radiation oncology [38], highlighting the growing role of interpretable models in clinical decision-making.

Future work should focus on external validation in multicenter cohorts to increase generalizability, integrate additional modalities such as genomics and PET imaging, and develop real-time prediction tools for clinical deployment. Moreover, a deeper exploration of the biological mechanisms underlying the association between RP and improved survival is warranted. Standardizing image preprocessing parameters (e.g., LoG sigma values) and validating SHAP explanations with clinician expertise will be essential to ensure reproducibility and clinical applicability.

## 5. Conclusions

This study developed Ensemble Stacking models centered on radiomics to predict radiation pneumonitis (RP) risk and three-year survival in NSCLC patients following VMAT, incorporating explainable artificial intelligence (XAI). The models achieved strong predictive performance, with an AUC of 0.91 for RP prediction and 0.98 for survival prediction. SHAP analysis identified V40 Firstorder_Min and V10 Wavelet Firstorder_Min as key features, confirming that radiomics provides superior predictive power compared with clinical and DVH features. SHAP-identified radiomic features, integrated with AI-driven tools, offer potential biomarkers to guide precise radiotherapy decisions in patients with NSCLC.

Key contributions include the demonstrated superiority of LASSO over Boruta-SHAP for feature selection, the added value of filtered radiomic features in enhancing RP prediction, the improved interpretability provided by XAI, and the novel finding of a paradoxical association between RP occurrence and improved survival (*p* = 0.024). Collectively, these results align with the principles of precision medicine, offering guidance for individualized treatment planning to minimize complications and improve survival, thereby providing a reliable foundation for clinical prognostic management.

The observed association between RP occurrence and improved survival suggests that mild RP may serve as a potential surrogate indicator of the radiation-induced tumor response; however, this hypothesis requires further biological validation. With additional external validation, the proposed model could be integrated into AI-assisted treatment planning systems (TPSs) to support personalized radiotherapy strategies in patients with NSCLC.

## Figures and Tables

**Table 1:** Clinical characteristics of patients with and without RP occurrence, including categories and percentages.

Characteristics	Total n = 168 (100%)	RP^2+^ n = 47 (28%)	Non-RP n = 121 (72%)	*p*-Value
Age (years)				0.345
Mean ± SD (Range)	68.6 ± 11.1 (39–96)	69.6 ± 10.8 (43–90)	67.8 ± 11.1 (39–96)	
BMI				0.174
Mean ± SD (Range)	23.6 ± 4.5 (9.6–40.3)	24.4 ± 4.6 (9.6–34.4)	23.4 ± 4.3 (13.7–40.3)	
Gender (%)				0.315
Male	123 (73)	37 (22)	86 (51)	
Female	45 (27)	10 (6)	35 (21)	
T-stage (%)				0.686
T1	31 (18)	9 (5)	22 (13)	
T2	54 (32)	18 (11)	36 (21)	
T3	35 (21)	9 (5)	26 (16)	
T4	48 (29)	11 (7)	37 (22)	
N-stage (%)				0.422
N0	54 (32)	14 (8)	40 (24)	
N1	21 (13)	6 (3)	15 (9)	
N2	63 (37)	15 (9)	48 (29)	
N3	30 (18)	12 (7)	18 (11)	
Chemotherapy (%)				0.412
YES	142 (85)	38 (23)	104 (62)	
NO	26 (15)	9 (5)	17 (10)	

Abbreviation: SD: Standard Deviation, BMI: Body Mass Index, T-stage: Tumor stage, N-stage: Node stage.

**Table 2:** Clinical characteristics of patients stratified by survival status, including categories and percentages.

Characteristics	Total n = 118 (100%)	Survival n = 84 (71%)	Death n = 34 (29%)	*p*-Value
Age (years)				0.595
Mean ± SD (Range)	68.8 ± 11.6 (39–96)	69.2 ± 10.9 (39–96)	67.9 ± 13.2 (42–96)	
BMI				0.022
Mean ± SD (Range)	23.5 ± 4.5 (9.6–40.3)	24.1 ± 4.6 (9.6–40.3)	22.0 ± 3.8 (13.9–30.4)	
Gender (%)				0.170
Male	35 (30)	28 (24)	7 (6)	
Female	83 (70)	56 (47)	27 (23)	
T-stage (%)				0.134
T1	24 (20)	20 (17)	4 (4)	
T2	42 (36)	32 (27)	10 (8)	
T3	25 (21)	17 (14)	8 (7)	
T4	27 (23)	15 (13)	12 (10)	
N-stage (%)				0.144
N0	39 (33)	33 (28)	6 (5)	
N1	15 (13)	9 (7)	6 (5)	
N2	42 (36)	27 (23)	15 (13)	
N3	22 (19)	15 (13)	7 (6)	
Chemotherapy (%)				0.224
YES	101 (86)	10 (8)	7 (6)	
NO	17 (14)	74 (63)	27 (23)	

Abbreviation: SD: Standard Deviation, BMI: Body Mass Index, T-stage: Tumor stage, N-stage: Node stage.

**Table 3:** Ensemble stacking models for RP and survival prediction constructed using LASSO-selected single- and multi-modal feature subsets.

RP_Ensemble Stacking							
Feature Subsets	AUC (95% CI)	ACC (95% CI)	NPV (95% CI)	Precision (95% CI)	Sensitivity (95% CI)	Specificity (95% CI)	F1 Score (95% CI)
F_C_	0.68 (0.44–0.87)	0.64 (0.44–0.81)	0.77 (0.53–0.95)	0.40 (0.10–0.75)	0.51 (0.14–0.88)	0.69 (0.46–0.89)	0.44 (0.13–0.71)
F_DVH_	0.54 (0.30–0.80)	0.60 (0.41–0.78)	0.79 (0.55–1.00)	0.39 (0.14–0.67)	0.64 (0.29–1.00)	0.58 (0.36–0.81)	0.47 (0.21–0.73)
F_R_O_	0.39 (0.18–0.60)	0.56 (0.38–0.71)	0.70 (0.50–0.89)	0.27 (0.00–0.57)	0.30 (0.00–0.63)	0.66 (0.45–0.84)	0.27 (0.00–0.50)
F_R_LoG_	0.57 (0.33–0.81)	0.68 (0.53–0.82)	0.76 (0.58–0.92)	0.44 (0.11–0.80)	0.40 (0.09–0.75)	0.79 (0.62–0.95)	0.41 (0.12–0.67)
F_R_W_	0.70 (0.49–0.88)	0.74 (0.59–0.88)	0.80 (0.64–0.95)	0.55 (0.25–0.89)	0.50 (0.20–0.80)	0.83 (0.67–0.96)	0.51 (0.22–0.77)
**F_R_**	**0.91** **(0.78–1.00)**	**0.89** **(0.74–1.00)**	**1.00** **(1.00–1.00)**	**0.73** **(0.43–1.00)**	**1.00** **(1.00–1.00)**	**0.84** **(0.67–1.00)**	**0.83** **(0.60–1.00)**
F_C+DVH_	0.42 (0.19–0.70)	0.45 (0.26–0.63)	0.68 (0.38–0.92)	0.27 (0.07–0.53)	0.51 (0.14–0.89)	0.43 (0.21–0.67)	0.34 (0.11–0.59)
F_C+R_	0.91 (0.78–1.00)	0.81 (0.67–0.96)	0.85 (0.67–1.00)	0.71 (0.33–1.00)	0.62 (0.25–1.00)	0.89 (0.73–1.00)	0.65 (0.31–0.91)
F_DVH+R_	0.87 (0.73–0.98)	0.78 (0.63–0.93)	0.81 (0.62–0.95)	0.67 (0.25–1.00)	0.49 (0.14–0.86)	0.90 (0.74–1.00)	0.54 (0.20–0.83)
F_C+DVH+R_	0.81 (0.62–0.96)	0.78 (0.63–0.93)	0.81 (0.62–0.95)	0.67 (0.25–1.00)	0.49 (0.14–0.86)	0.90 (0.74–1.00)	0.54 (0.20–0.83)
**Survival_Ensemble Stacking**							
**Feature Subsets**	**AUC** **(95% CI)**	**ACC** **(95% CI)**	**NPV** **(95% CI)**	**Precision (95% CI)**	**Sensitivity (95% CI)**	**Specificity (95% CI)**	**F1 Score** **(95% CI)**
F_C_	0.74 (0.52–0.92)	0.72 (0.54–0.88)	0.82 (0.61–1.00)	0.51 (0.17–0.86)	0.58 (0.20–1.00)	0.78 (0.56–0.94)	0.53 (0.18–0.80)
F_DVH_	0.50 (0.21–0.80)	0.71 (0.50–0.88)	0.78 (0.56–0.95)	0.49 (0.00–1.00)	0.43 (0.00–0.83)	0.82 (0.63–1.00)	0.44 (0.00–0.75)
F_R_O_	0.83 (0.62–1.00)	0.71 (0.54–0.88)	0.86 (0.65–1.00)	0.50 (0.18–0.83)	0.72 (0.33–1.00)	0.71 (0.47–0.92)	0.58 (0.25–0.84)
F_R_LoG_	0.85 (0.68–0.99)	0.75 (0.58–0.92)	0.83 (0.63–1.00)	0.57 (0.17–1.00)	0.58 (0.17–1.00)	0.82 (0.63–1.00)	0.55 (0.18–0.82)
F_R_W_	0.92 (0.75–1.00)	0.75 (0.54–0.92)	0.87 (0.67–1.00)	0.55 (0.20–0.89)	0.72 (0.33–1.00)	0.76 (0.56–0.94)	0.61 (0.27–0.88)
**F_R_**	**0.9** **7** **(0.8** **8–1.00)**	**0.9** **2** **(0.** **79–1.00)**	**1.00** **(** **1.00–1.00)**	**0.77** **(** **0.45–1.00)**	**1.00** **(** **1.00–1.00)**	**0.88** **(0.** **71–1.00)**	**0.** **86** **(0.6** **2–1.00)**
F_C+DVH_	0.74 (0.50–0.92)	0.63 (0.42–0.79)	0.75 (0.50–0.94)	0.37 (0.00–0.75)	0.43 (0.00–0.83)	0.70 (0.50–0.92)	0.39 (0.00–0.67)
F_C+R_	0.96 (0.86–1.00)	0.88 (0.75–1.00)	0.89 (0.72–1.00)	0.84 (0.50–1.00)	0.73 (0.33–1.00)	0.94 (0.81–1.00)	0.76 (0.40–1.00)
F_DVH+R_	0.92 (0.78–1.00)	0.83 (0.67–0.96)	0.94 (0.79–1.00)	0.66 (0.33–1.00)	0.86 (0.50–1.00)	0.82 (0.63–1.00)	0.73 (0.40–0.94)
F_C+DVH+R_	0.93 (0.80–1.00)	0.88 (0.75–1.00)	0.89 (0.72–1.00)	0.84 (0.50–1.00)	0.73 (0.33–1.00)	0.94 (0.81–1.00)	0.76 (0.40–1.00)

Abbreviation: DVH: Dose-volume histogram, SHAP: SHapley Additive exPlanations, RP: Radiation Pneumonitis, F: Feature subset, C: Clinical, O: Original image, W: Wavelet, LoG: Laplacian of Gaussian, LR: Logistic Regression, RF: Random Forest, KNN: K-Nearest Neighbors, SVM: Support Vector Machine, XGBoost: eXtreme Gradient Boosting, AUC: Area Under the ROC curve, ROC: Receiver Operating Characteristic, ACC: Accuracy, NPV: Negative Predictive Value, LoG: Laplacian of Gaussian, W: Wavelet filter, R: Radiomics, CI: Confidence Interval.

## Data Availability

The datasets generated and/or analyzed during the current study are not publicly available due to institutional ethical restrictions and patient privacy regulations but are available from the corresponding authors (Pei-Ju Chao, pjchao99@gmail.com; Yu-Wei Lin, marklin1108@gmail.com) upon reasonable request and with appropriate ethical approval from the Kaohsiung Veterans General Hospital Institutional Review Board (IRB No. KSVGH25-CT1-06). Supporting data, including feature extraction scripts and model performance metrics, are provided in the Supplementary Materials.

## Supplementary Materials

The following supporting information can be downloaded at: https://www.mdpi.com/article/10.3390/life15111753/s1, Supplementary Table S1 summarizes feature selection results for 10 subsets in RP prediction using Boruta-SHAP and LASSO. Supplementary Table S2 summarizes feature selection results for 10 subsets in survival prediction using Boruta-SHAP and LASSO. Supplementary Table S3 details RP prediction models with LASSO-selected subsets, including performance metrics (AUC, ACC, NPV, Precision, Sensitivity, Specificity, F1-score). Supplementary Table S4 details survival prediction models with LASSO-selected subsets and metrics. Supplementary Table S5 details RP prediction models with Boruta-SHAP-selected subsets and metrics. Supplementary Table S6 details survival prediction models with Boruta-SHAP-selected subsets and metrics. Supplementary Table S7 shows the statistical association between RP occurrence and survival status. Supplementary Table S8 lists the top five selected radiomics features from single-feature subsets using Boruta-SHAP and LASSO.

## Abbreviations

ACC, Accuracy; AI, Artificial Intelligence; ANOVA, Analysis of Variance; AUC, Area Under the Curve; CT, Computed Tomography; DICOM, Digital Imaging and Communications in Medicine; DVH, Dose–Volume Histogram; FC, Clinical Feature Subset; FDVH, Dose–Volume Histogram Feature Subset; FR, Filtered Radiomics Feature Subset; F1, F1-score; GLDM, Gray Level Dependence Matrix; GLCM, Gray Level Co-occurrence Matrix; GLRLM, Gray Level Run Length Matrix; GLSZM, Gray Level Size Zone Matrix; IRB, Institutional Review Board; KNN, K-Nearest Neighbors; LASSO, Least Absolute Shrinkage and Selection Operator; O, Original image; W, Wavelet; LoG, Laplacian of Gaussian; LR, Logistic Regression; MLD, Mean Lung Dose; ML, Machine Learning; NGTDM, Neighboring Gray Tone Difference Matrix; NPV, Negative Predictive Value; NSCLC, Non–Small Cell Lung Cancer; OS, Overall Survival; PTV, Planning Target Volume; Pre., Precision; RF, Random Forest; RP, Radiation Pneumonitis; RT, Radiation Therapy; RTOG, Radiation Therapy Oncology Group; Sen., Sensitivity; SHAP, SHapley Additive exPlanations; SMOTE, Synthetic Minority Oversampling Technique; Spe., Specificity; SVM, Support Vector Machine; TPS, Treatment Planning System; V5/V10/V20/V40, Lung Volume Receiving ≥5/10/20/40 Gy; VMAT, Volumetric Modulated Arc Therapy; XAI, Explainable Artificial Intelligence; XGBoost, eXtreme Gradient Boosting.
