# Supplementary material for: Using Radiomics and Explainable Ensemble Learning to Predict Radiation Pneumonitis and Survival in NSCLC Patients Post-VMAT
Source: Life (Basel). 2025 Nov 14;15(11):1753. doi: 10.3390/life15111753 (PMC12653653; doi:10.3390/life15111753)
Supplement: Supplementary file 1 [file life-15-01753-s001.zip › life-3906386-supplementary.pdf]

Supplementary

Table S1. Feature selection results of 10 feature subsets for RP prediction using Boruta-SHAP and LASSO methods

Feature Subsets	Numbers	Boruta-SHAP	LASSO
F_C	7	1	2
F_{DVH}	9	2	1
F_{R_O}	23	5	6
F_{R_LoG}	150	12	14
F_{R_W}	442	11	28
F_R	615	17	35
$F_{C,\ DVH}$	16	3	3
$F_{C,\ R}$	622	18	34
$F_{DVH,\ R}$	624	15	35
$F_{C,\ DVH,\ R}$	631	18	32

Abbreviation: LASSO: Least Absolute Shrinkage and Selection Operator, F: Feature subset, C: Clinical, DVH: Dose-Volume Histogram, R: Radiomics, O: Original, W: Wavelet, LoG: Laplacian of Gaussian

Table S2. Feature selection results of 10 feature subsets for survival prediction using Boruta-SHAP and LASSO methods

Feature Subsets	Numbers	Boruta-SHAP	LASSO
F_C	7	2	1
F_{DVH}	9	1	3
F_{R_O}	79	11	9
F_{R_LoG}	314	15	13
F_{R_W}	778	19	24
F_R	1171	18	28
$F_{C,\ DVH}$	16	3	3
$F_{C,\ R}$	1178	18	25
$F_{DVH,\ R}$	1180	19	28
$F_{C,\ DVH,\ R}$	1187	16	24

Abbreviation: LASSO: Least Absolute Shrinkage and Selection Operator, F: Feature subset, C: Clinical, DVH: Dose-Volume Histogram, R: Radiomics, O: Original, W: Wavelet, LoG: Laplacian of Gaussian

Table S3. RP prediction models constructed with LASSO–selected feature subsets

Model	AUC	ACC	NPV	Pre.	Sen.	Spe.	F1	AUC	ACC	NPV	Pre.	Sen.	Spe.	F1
				**F_{C}**							**F_{DVH}**			
LR	0.46	0.47	0.67	0.25	0.40	0.50	0.31	0.51	0.53	0.75	0.33	0.60	0.50	0.43
RF	0.50	0.53	0.70	0.29	0.40	0.58	0.33	0.50	0.50	0.73	0.32	0.60	0.58	0.40
SVM	0.48	0.53	0.70	0.29	0.40	0.58	0.33	0.49	0.50	0.73	0.32	0.60	0.46	0.41
XGBoost	0.46	0.50	0.69	0.28	0.50	0.46	0.36	0.45	0.47	0.71	0.30	0.60	0.42	0.40
KNN	0.54	0.59	0.73	0.33	0.40	0.67	0.36	0.49	0.50	0.67	0.23	0.30	0.58	0.26
				**F_{R_O}**							**F_{R_LoG}**			
LR	0.46	0.47	0.67	0.25	0.40	0.50	0.31	0.74	0.62	0.79	0.40	0.60	0.63	0.48
RF	0.50	0.53	0.70	0.29	0.40	0.58	0.33	0.60	0.74	0.78	0.57	0.40	0.88	0.47
SVM	0.48	0.53	0.70	0.29	0.40	0.58	0.33	0.71	0.65	0.77	0.42	0.50	0.71	0.46
XGBoost	0.46	0.50	0.69	0.28	0.50	0.46	0.36	0.66	0.77	0.79	0.67	0.40	0.92	0.50
KNN	0.54	0.59	0.73	0.33	0.40	0.67	0.36	0.70	0.62	0.82	0.41	0.70	0.58	0.52
				**F_{R_W}**							**F_{R}**			
LR	0.73	0.68	0.78	0.46	0.50	0.75	0.48	0.82	0.74	0.86	0.54	0.70	0.75	0.61
RF	0.67	0.65	0.73	0.38	0.30	0.79	0.33	0.72	0.71	0.77	0.50	0.40	0.83	0.44
SVM	0.74	0.68	0.84	0.47	0.70	0.67	0.56	0.81	0.74	0.80	0.56	0.50	0.83	0.53
XGBoost	0.73	0.74	0.83	0.55	0.60	0.79	0.57	0.76	0.74	0.80	0.56	0.50	0.83	0.53
KNN	0.70	0.62	0.82	0.41	0.70	0.58	0.52	0.83	0.79	0.91	0.62	0.80	0.79	0.70
				**F_{C+DVH}**							**F_{C+R}**			
LR	0.47	0.47	0.67	0.25	0.40	0.50	0.31	0.85	0.77	0.86	0.58	0.70	0.79	0.64
RF	0.52	0.65	0.77	0.42	0.50	0.71	0.46	0.66	0.71	0.77	0.50	0.40	0.83	0.44
SVM	0.47	0.47	0.69	0.28	0.50	0.46	0.36	0.82	0.77	0.81	0.63	0.50	0.88	0.56
XGBoost	0.51	0.50	0.69	0.27	0.40	0.54	0.32	0.71	0.77	0.83	0.60	0.60	0.83	0.60
KNN	0.47	0.47	0.67	0.25	0.40	0.50	0.31	0.85	0.74	0.90	0.53	0.80	0.71	0.64
				**F_{DVH+R}**							**$F_{C+DVH+R}$**			
LR	0.83	0.74	0.86	0.54	0.70	0.75	0.61	0.81	0.77	0.86	0.58	0.70	0.79	0.64
RF	0.76	0.74	0.80	0.56	0.50	0.83	0.53	0.73	0.74	0.78	0.57	0.40	0.88	0.47
SVM	0.82	0.79	0.84	0.67	0.60	0.88	0.63	0.81	0.74	0.78	0.57	0.40	0.88	0.47
XGBoost	0.77	0.71	0.79	0.50	0.50	0.79	0.50	0.75	0.71	0.77	0.50	0.40	0.83	0.44
KNN	0.82	0.79	0.91	0.62	0.80	0.79	0.70	0.84	0.71	0.94	0.50	0.90	0.63	0.64

Abbreviation: DVH: Dose-volume histogram, LASSO: Least Absolute Shrinkage and Selection Operator, RP: Radiation Pneumonitis, F: Feature subset, C: Clinical, LR: Logistic Regression, RF: Random Forest, KNN: K-Nearest Neighbors, SVM: Support Vector Machine, XGBoost: eXtreme Gradient Boosting, AUC: Area Under the ROC curve, ROC: Receiver Operating Characteristic, ACC: Accuracy, NPV: Negative Predictive Value, Prec.: Precision, Sen.: Sensitivity, Spe.: Specificity, F1: F1-score, O: Original image, LoG: Laplacian of Gaussian, W: Wavelet filter, R: Radiomics

Table S4. RP prediction models constructed with Boruta-SHAP–selected feature subsets

				F_C							F_{DVH}			
Model	AUC	ACC	NPV	Pre.	Sen.	Spe.	F1	AUC	ACC	NPV	Pre.	Sen.	Spe.	F1
LR	0.52	0.59	0.75	0.36	0.50	0.63	0.52	0.48	0.50	0.77	0.33	0.70	0.42	0.45
RF	0.51	0.56	0.71	0.31	0.40	0.63	0.35	0.33	0.53	0.65	0.13	0.10	0.71	0.11
SVM	0.52	0.56	0.70	0.27	0.30	0.67	0.29	0.52	0.44	0.73	0.30	0.70	0.33	0.42
XGBoost	0.55	0.56	0.70	0.27	0.30	0.67	0.29	0.48	0.62	0.72	0.33	0.30	0.75	0.32
KNN	0.52	0.59	0.73	0.33	0.40	0.67	0.37	0.49	0.59	0.73	0.33	0.40	0.67	0.36
Ensemble	0.58	0.62	0.74	0.36	0.40	0.71	0.38	0.54	0.62	0.70	0.29	0.20	0.79	0.24
				F_{R_O}							F_{R_LoG}			
LR	0.50	0.71	0.71	0.00	0.00	1.00	0.00	0.69	0.65	0.77	0.42	0.50	0.71	0.46
RF	0.61	0.59	0.75	0.36	0.50	0.63	0.42	0.72	0.77	0.81	0.63	0.50	0.88	0.56
SVM	0.54	0.62	0.76	0.39	0.50	0.67	0.44	0.54	0.68	0.76	0.44	0.40	0.79	0.42
XGBoost	0.52	0.53	0.72	0.31	0.50	0.54	0.39	0.73	0.77	0.79	0.67	0.40	0.92	0.50
KNN	0.49	0.47	0.67	0.25	0.40	0.50	0.31	0.60	0.62	0.74	0.36	0.40	0.71	0.38
Ensemble	0.69	0.62	0.79	0.40	0.60	0.63	0.48	0.75	0.82	0.85	0.75	0.60	0.92	0.67
				F_{R_W}							F_R			
LR	0.69	0.62	0.87	0.42	0.80	0.54	0.55	0.72	0.59	0.78	0.38	0.60	0.58	0.46
RF	0.68	0.65	0.83	0.44	0.70	0.63	0.54	0.74	0.65	0.80	0.43	0.60	0.67	0.50
SVM	0.70	0.62	0.87	0.42	0.80	0.54	0.55	0.65	0.62	0.72	0.33	0.30	0.75	0.32
XGBoost	0.58	0.56	0.75	0.38	0.80	0.46	0.52	0.76	0.71	0.82	0.50	0.60	0.75	0.55
KNN	0.68	0.59	0.92	0.41	0.90	0.46	0.56	0.72	0.59	0.78	0.38	0.60	0.58	0.46
Ensemble	0.74	0.71	0.89	0.50	0.80	0.67	0.62	0.83	0.77	0.83	0.60	0.60	0.83	0.60
				F_{C+DVH}							F_{C+R}			
LR	0.52	0.59	0.75	0.36	0.50	0.63	0.42	0.60	0.59	0.75	0.36	0.50	0.63	0.42
RF	0.45	0.56	0.68	0.22	0.20	0.71	0.21	0.69	0.71	0.79	0.50	0.50	0.79	0.50
SVM	0.47	0.44	0.65	0.24	0.40	0.56	0.30	0.60	0.68	0.78	0.46	0.50	0.75	0.48
XGBoost	0.43	0.62	0.70	0.29	0.20	0.79	0.24	0.73	0.68	0.76	0.44	0.40	0.79	0.42
KNN	0.58	0.65	0.77	0.42	0.50	0.71	0.46	0.63	0.56	0.71	0.31	0.40	0.63	0.35
Ensemble	0.60	0.62	0.76	0.39	0.50	0.67	0.44	0.80	0.74	0.80	0.56	0.50	0.83	0.53
				F_{DVH+R}							$F_{C+DVH+R}$			
LR	0.66	0.62	0.79	0.40	0.60	0.63	0.48	0.68	0.62	0.76	0.39	0.50	0.67	0.44
RF	0.74	0.74	0.80	0.56	0.50	0.83	0.53	0.71	0.65	0.80	0.43	0.60	0.67	0.50
SVM	0.69	0.62	0.76	0.39	0.50	0.67	0.44	0.68	0.68	0.78	0.46	0.50	0.75	0.48
XGBoost	0.75	0.65	0.80	0.43	0.60	0.67	0.50	0.73	0.62	0.79	0.40	0.60	0.63	0.48
KNN	0.63	0.62	0.82	0.41	0.70	0.58	0.52	0.64	0.62	0.76	0.39	0.50	0.67	0.44
Ensemble	0.78	0.77	0.83	0.60	0.60	0.83	0.60	0.74	0.68	0.78	0.45	0.50	0.75	0.48

Abbreviation: DVH: Dose-volume histogram, LASSO: Least Absolute Shrinkage and Selection Operator, RP: Radiation Pneumonitis, F: Feature subset, C: Clinical, LR: Logistic Regression, RF: Random Forest, KNN: K-Nearest Neighbors, SVM: Support Vector Machine, XGBoost: eXtreme Gradient Boosting, AUC: Area Under the ROC curve, ROC: Receiver Operating Characteristic, ACC: Accuracy, NPV: Negative Predictive Value, Prec.: Precision, Sen.: Sensitivity, Spe.: Specificity, F1: F1-score, O: Original image, LoG: Laplacian of Gaussian, W: Wavelet filter, R: Radiomics

Table S5. Survival prediction models constructed with LASSO–selected feature subsets

				F_C							F_{DVH}			
Model	**AUC**	**ACC**	**NPV**	**Pre.**	**Sen.**	**Spe.**	**F1**	**AUC**	**ACC**	**NPV**	**Pre.**	**Sen.**	**Spe.**	**F1**
LR	0.79	0.71	0.86	0.50	0.71	0.71	0.59	0.47	0.58	0.73	0.33	0.43	0.65	0.38
RF	0.66	0.67	0.91	0.46	0.86	0.58	0.60	0.54	0.58	0.71	0.29	0.29	0.71	0.29
SVM	0.62	0.46	1.00	0.35	1.00	0.24	0.52	0.46	0.50	0.67	0.22	0.29	0.59	0.25
XGBoost	0.65	0.63	0.83	0.42	0.71	0.59	0.53	0.49	0.63	0.72	0.33	0.29	0.77	0.31
KNN	0.62	0.63	0.75	0.38	0.43	0.71	0.40	0.44	0.50	0.67	0.22	0.29	0.29	0.25
				F_{R_O}							F_{R_LoG}			
LR	0.94	0.92	1.00	0.78	1.00	0.88	0.88	0.86	0.67	0.80	0.44	0.57	0.71	0.50
RF	0.85	0.75	0.92	0.55	0.86	0.71	0.67	0.80	0.67	0.77	0.43	0.43	0.77	0.43
SVM	0.94	0.79	0.93	0.60	0.86	0.77	0.71	0.85	0.75	0.79	0.60	0.43	0.88	0.50
XGBoost	0.93	0.83	1.00	0.64	1.00	0.77	0.78	0.77	0.71	0.78	0.50	0.43	0.82	0.46
KNN	0.89	0.83	1.00	0.64	1.00	0.77	0.78	0.80	0.83	0.93	0.67	0.86	0.82	0.75
				F_{R_W}							F_R			
LR	0.88	0.83	0.93	0.67	0.86	0.82	0.75	0.86	0.83	0.88	0.71	0.71	0.88	0.71
RF	0.95	0.79	0.93	0.60	0.86	0.77	0.71	0.96	0.88	0.94	0.75	0.86	0.88	0.80
SVM	0.94	0.79	0.88	0.63	0.71	0.82	0.67	0.91	0.83	0.93	0.67	0.86	0.82	0.75
XGBoost	0.96	0.83	0.93	0.67	0.86	0.82	0.75	0.93	0.88	0.94	0.75	0.86	0.88	0.80
KNN	0.96	0.83	1.00	0.64	1.00	0.77	0.78	0.90	0.79	0.93	0.60	0.86	0.77	0.71
				F_{C+DVH}							F_{C+R}			
LR	0.72	0.67	0.77	0.43	0.43	0.77	0.43	0.92	0.88	0.94	0.75	0.86	0.88	0.80
RF	0.68	0.58	0.71	0.29	0.29	0.71	0.29	0.96	0.83	1.00	0.64	1.00	0.77	0.78
SVM	0.74	0.71	0.81	0.50	0.57	0.77	0.53	0.92	0.79	0.83	0.67	0.57	0.88	0.62
XGBoost	0.65	0.63	0.72	0.33	0.29	0.77	0.31	0.96	0.88	1.00	0.70	1.00	0.82	0.82
KNN	0.74	0.67	0.80	0.44	0.57	0.71	0.50	0.90	0.79	1.00	0.58	1.00	0.71	0.74
				F_{DVH+R}							$F_{C+DVH+R}$			
LR	0.74	0.71	0.75	0.50	0.29	0.88	0.36	0.95	0.88	1.00	0.70	1.00	0.82	0.82
RF	0.95	0.88	0.94	0.75	0.86	0.88	0.80	0.97	0.83	0.93	0.67	0.86	0.82	0.75
SVM	0.89	0.83	0.88	0.71	0.71	0.88	0.71	0.92	0.83	0.84	0.80	0.57	0.94	0.67
XGBoost	0.97	0.82	1.00	0.78	1.00	0.88	0.88	0.94	0.88	1.00	0.70	1.00	0.82	0.82
KNN	0.87	0.75	0.92	0.55	0.86	0.71	0.67	0.92	0.75	1.00	0.54	1.00	0.65	0.70

Abbreviation: DVH: Dose-volume histogram, LASSO: Least Absolute Shrinkage and Selection Operator, F: Feature subset, C: Clinical, LR: Logistic Regression, RF: Random Forest, KNN: K-Nearest Neighbors, SVM: Support Vector Machine, XGBoost: eXtreme Gradient Boosting, AUC: Area Under the ROC curve, ROC: Receiver Operating Characteristic, ACC: Accuracy, NPV: Negative Predictive Value, Prec.: Precision, Sen.: Sensitivity, Spe.: Specificity, F1: F1-score, O: Original image, LoG: Laplacian of Gaussian, W: Wavelet filter, R: Radiomics

Table S6. Survival prediction models constructed with Boruta-SHAP–selected feature subsets

				F_C							F_{DVH}			
Model	AUC	ACC	NPV	Pre.	Sen.	Spe.	F1	AUC	ACC	NPV	Pre.	Sen.	Spe.	F1
LR	0.50	0.71	0.71	0.00	0.00	1.00	0.00	0.50	0.71	0.71	0.00	0.00	1.00	0.00
RF	0.65	0.67	0.85	0.46	0.72	0.65	0.56	0.50	0.42	0.64	0.23	0.43	0.41	0.30
SVM	0.62	0.54	0.75	0.33	0.58	0.53	0.42	0.54	0.58	0.75	0.33	0.57	0.53	0.42
XGBoost	0.58	0.54	0.75	0.33	0.57	0.53	0.42	0.49	0.63	0.72	0.33	0.29	0.77	0.31
KNN	0.45	0.42	0.64	0.23	0.43	0.41	0.30	0.51	0.42	0.64	0.23	0.43	0.41	0.30
Ensemble	0.69	0.71	0.86	0.50	0.71	0.71	0.59	0.63	0.67	0.77	0.43	0.43	0.77	0.43
				F_{R_O}							F_{R_LoG}			
LR	0.91	0.83	1.00	0.64	1.00	0.77	0.78	0.87	0.71	0.86	0.50	0.71	0.71	0.59
RF	0.88	0.75	0.92	0.55	0.86	0.71	0.67	0.82	0.79	0.93	0.60	0.86	0.77	0.71
SVM	0.91	0.83	1.00	0.64	1.00	0.77	0.78	0.87	0.79	1.00	0.58	1.00	0.71	0.74
XGBoost	0.83	0.79	1.00	0.58	1.00	0.71	0.74	0.80	0.75	0.87	0.56	0.71	0.77	0.63
KNN	0.87	0.79	0.93	0.60	0.86	0.77	0.71	0.74	0.67	0.85	0.46	0.71	0.65	0.56
Ensemble	0.93	0.83	0.93	0.67	0.86	0.62	0.75	0.92	0.88	0.93	0.67	0.86	0.82	0.75
				F_{R_W}							F_R			
LR	0.92	0.88	0.94	0.75	0.86	0.88	0.80	0.77	0.75	0.87	0.56	0.71	0.77	0.63
RF	0.93	0.83	0.93	0.67	0.86	0.82	0.75	0.91	0.83	0.93	0.67	0.86	0.82	0.75
SVM	0.93	0.83	1.00	0.64	1.00	0.77	0.78	0.88	0.83	1.00	0.64	1.00	0.77	0.78
XGBoost	0.87	0.79	0.93	0.60	0.86	0.77	0.71	0.91	0.75	0.92	0.55	0.86	0.71	0.67
KNN	0.86	0.79	0.93	0.60	0.86	0.77	0.71	0.86	0.79	0.93	0.60	0.86	0.77	0.71
Ensemble	0.93	0.88	1.00	0.70	1.00	0.82	0.82	0.94	0.88	0.94	0.75	0.86	0.88	0.80
				F_{C+DVH}							F_{C+R}			
LR	0.50	0.71	0.71	0.00	0.00	1.00	0.00	0.90	0.75	0.92	0.55	0.86	0.71	0.67
RF	0.57	0.58	0.71	0.29	0.29	0.71	0.29	0.93	0.79	0.93	0.60	0.86	0.77	0.71
SVM	0.55	0.50	0.73	0.31	0.57	0.47	0.40	0.84	0.67	0.85	0.46	0.71	0.65	0.56
XGBoost	0.58	0.63	0.75	0.38	0.43	0.71	0.40	0.92	0.79	0.93	0.60	0.86	0.77	0.71
KNN	0.57	0.58	0.73	0.33	0.43	0.65	0.38	0.82	0.75	0.92	0.55	0.86	0.71	0.67
Ensemble	0.59	0.71	0.78	0.50	0.43	0.82	0.46	0.94	0.83	0.93	0.67	0.86	0.82	0.75
				F_{DVH+R}							$F_{C+DVH+R}$			
LR	0.92	0.79	0.88	0.63	0.71	0.82	0.67	0.80	0.75	1.00	0.54	1.00	0.65	0.70
RF	0.92	0.83	0.93	0.68	0.86	0.82	0.75	0.81	0.75	0.92	0.55	0.86	0.71	0.67
SVM	0.82	0.75	0.87	0.56	0.71	0.77	0.63	0.77	0.75	0.87	0.56	0.71	0.77	0.63
XGBoost	0.91	0.83	1.00	0.64	1.00	0.77	0.78	0.87	0.79	0.93	0.60	0.86	0.77	0.71
KNN	0.82	0.79	0.88	0.63	0.71	0.82	0.67	0.85	0.75	0.92	0.55	0.86	0.71	0.67
Ensemble	0.93	0.88	1.00	0.70	1.00	0.82	0.82	0.92	0.83	0.93	0.67	0.86	0.82	0.75

Abbreviation: DVH: Dose-volume histogram, SHAP: SHapley Additive exPlanations, F: Feature subset, C: Clinical, LR: Logistic Regression, RF: Random Forest, KNN: K-Nearest Neighbors, SVM: Support Vector Machine, XGBoost: eXtreme Gradient Boosting, AUC: Area Under the ROC curve, ROC: Receiver Operating Characteristic, ACC: Accuracy, NPV: Negative Predictive Value, Prec.: Precision, Sen.: Sensitivity, Spe.: Specificity, F1: F1-score, O: Original image, LoG: Laplacian of Gaussian, W: Wavelet filter, R: Radiomics

Table S7. Statistical association between RP occurrence and survival status

Characteristics	**Total n = 118 (100%)**	**Survival n = 84 (71%)**	**Death n = 34 (29%)**	**p-value**
**RP** (%)				0.024
NO	83 (86)	54 (46)	29 (25)	
YES	35 (14)	30 (25)	5 (4)	

Abbreviation: SD: Standard Deviation, BMI: Body Mass Index, T-stage: Tumor stage, N-stage: Node stage

Table S8. Top five selected radiomics features from single-feature subsets using Boruta-SHAP and LASSO methods

**Feature Selection Methods**	**Radiomics Feature Subsets (F_R)**
**Boruta-SHAP**	1. Radiomics_V20_LoG-Sigma-1-5-mm-3D_Firstorder_90Percentile<br>2. Radiomics_V50_Wavelet-HLH_GLSZM_Zonepercentage<br>3. Radiomics_V50_Wavelet-HLH_NGTDM_Contrast<br>4. Radiomics_V30_LoG-Sigma-1-5-mm-3D_Firstorder_Skewness<br>5. Radiomics_V50_Wavelet-HLH_Firstorder_10Percentile
**LASSO**	1. Radiomics_V20_LoG-Sigma-1-0-mm-3D_Firstorder_Skewness<br>2. Radiomics_V50_Wavelet-LHL_GLSZM_Smallarealowgraylevelemphasis<br>3. Radiomics_V5_Wavelet-HHH_Firstorder_Maximum<br>4. Radiomics_V50_LoG-Sigma-3-mm-3D_GLCM_Maximumprobability<br>5. Radiomics_V50_Wavelet-HLH_NGTDM_Contrast

Abbreviation: RP: Radiation Pneumonitis, F: Feature subset, R: Radiomics, SHAP: SHapley Additive exPlanations, V: Volume, GLSZM: Gray Level Size Zone Matrix, NGTDM: Neighbouring Gray Tone Difference Matrix, LASSO: Least Absolute Shrinkage and Selection Operator, GLCM: Gray Level Co-occurrence Matrix
